# Attenuated neuronal and autonomic responses during error processing in anorexia nervosa

**DOI:** 10.1002/brb3.2235

**Published:** 2021-07-28

**Authors:** Stefanie Suttkus, Andy Schumann, Feliberto De la Cruz, Karl‐Jürgen Bär

**Affiliations:** ^1^ Lab for Autonomic Neuroscience Imaging and Cognition (LANIC) Department of Psychosomatic Medicine and Psychotherapy Jena University Jena Germany

**Keywords:** Anorexia nervosa, high‐rsolution functional MRI, skin conductance, response inhibition

## Abstract

**Introduction:**

Anorexia nervosa (AN) is a severe psychiatric illness with alarming mortality rates. Nevertheless, despite former and recent research results, the etiology of AN is still poorly understood. Of particular interest is that, despite exaggerated response control and increased perfectionism scores, patients with AN seem not to perform better that those unaffected in tasks that require inhibitory control. One reason might be aberrant processing of errors. The objective of our study was thus to obtain further insight into the pathopsychology of AN. We were particularly interested in neuronal and autonomic responses during error processing and their association with behavior.

**Methods:**

We analyzed 16 acute patients suffering from restrictive type AN and 21 healthy controls using functional magnetic resonance imaging (fMRI) with simultaneous physiological recordings during a Go/Nogo response inhibition task. Data were corrected for noise due to cardiac and respiratory influence.

**Results:**

Patients and controls had similarly successful response inhibition in Nogo trials. However, in failed Nogo trials, controls had significantly greater skin conductance responses (SCR) than in correct Nogo trials. Patients did not exhibit elevated SCR to errors. Furthermore, we found significantly increased neuronal responses, especially in the amygdala and hippocampus, in controls compared to patients during error trials. We also found significant positive correlations in controls but not in patients between Nogo performance and activation in the salience network core regions after errors.

**Conclusion:**

Acute restrictive type AN patients seem to lack neuronal and autonomic responses to errors that might impede a flexible behavior adaption.

## 1 INTRODUCTION

Anorexia nervosa (AN) is a severe psychiatric illness with alarming mortality rates, especially in the restrictive type of the eating disorder (Arcelus et al., 2011). The characteristics of AN patients are low bodyweight, starvation to prevent weight gain and an enormous fear of being or becoming fat. A core feature is the highly distorted body image and various irrational beliefs about their own body (Treasure et al., 2015; Zipfel et al., 2015). Patients suffering from AN are characterized by increased self‐control and impaired set‐shifting abilities (Geisler et al., 2017). Moreover, Buzzichelli et al. (2018) found high cognitive rigidity in AN patients and identified perfectionism as an essential mediator of the relationship between set‐shifting abilities and eating psychopathology.

Although being the subject of considerable research, the etiology of AN is still poorly understood. Despite current psychological interventions, the disorder has poor remission rates and high levels of relapse (Jagielska & Kacperska, 2017). However, grad electrophysiological investigations and functional magnetic resonance imaging (fMRI) have gradually provided insights into the neuronal abnormalities of AN. A major topic of investigation is inhibitory cognitive because patients tend to be over‐controlled and perfectionistic. For instance, Oberndorfer et al. (2011) applied a response inhibition task during fMRI. They found lower medial prefrontal cortex (PFC) activation with increased task demands in AN patients than in healthy controls. Interestingly, however, patients and controls did not differ in performance. In the same kind of task, Wierenga et al. (2014) likewise found no group difference in performance between AN patients and control subjects. However, during error processing, patients had less activation in medial frontal regions (Wierenga et al., 2014). In an electrophysiological study, Pieters et al. (2007) found evidence for distorted processing of errors in AN. They reported diminished brain responses to errors and error‐related negativity, accompanied by increased perfectionism scores in patients. It seems that AN patients recruit fewer cognitive resources in response to errors indicating reduced error monitoring. They manage to perform equally well as healthy controls due to effortful supervisory cognitive control supporting cognitive rigidity (Wierenga et al., 2014; Zastrow et al., 2009).

Response errors or other salient stimuli activate a fronto‐limbic system, which is also called salience network, including the anterior insula (aIN), dorsal anterior cingulate cortex (dACC) and the amygdala. Following salience detection, the salience network promotes brain network switching and initiates further processing steps (Goulden et al., 2014; Menon, 2011; Seeley et al., 2007). The intact ability to switch between networks is crucial for optimal brain function (Pedersen et al., 2018).

In patients with AN, salience network function seems to be affected (Bang et al., 2016; Menon, 2011; Phillips et al., 2003). For instance, Bang et al. (2016) investigated recovered AN patients using fMRI and an emotional conflict task. They found patients to exhibit attenuated BOLD responses in the amygdala and hippocampus during significant stimuli processing. Insula malfunctions in AN patients are also supported by reduced activity in the insula during visceral interoception in AN patients (Kerr et al., 2016).

This brain network also elicits physiological responses to salient stimuli that facilitate affective processing (Damasio, 2003; Schachter & Singer, 1962). For instance, skin electrical conductance increases due to the accumulation of sweat in response to salient emotional stimuli or cognitive demand (Köhler, et al., 2018; Schumann et al., 2018; Zhang et al., 2012). It is of interest, however, that these autonomic responses seem to be blunted in patients with AN. Tchanturia et al. (2007) found reduced affective reactions indicated by diminished skin conductance responses (SCR) after losses in an Iowa gambling task. They argue that impaired decision‐making is a consequence of lack of bodily alarm signals. However, it is still unclear how physiological reactions to errors are related to exaggerated response control and perfectionism in AN patients.

The objective of our study was to obtain further insight into the cognitive processing of AN. In particular, we were interested in neuronal and autonomic responses to errors. We used fMRI with simultaneous skin conductance recordings during a response inhibition task. According to previous findings, we stated the following hypotheses: (1) Patients and controls do not differ in behavioral performance during the task. (2) In AN patients, errors reduce SCR and (3) reduce BOLD activation in core regions of the salience network, i.e., dACC and aIN, compared to controls. (4) Brain activation and SCR are associated with task performance.

## 2 METHODS

### 2.1 Subjects

A total of 21 healthy controls (2 male, 19 female; age M = 26.29 years; SD = 6.9 years) were recruited from the local community and interviewed by a medical research assistant. Individuals with past or current drug use, any medical condition, neurological or psychiatric diseases and/or first‐degree relatives with Axis I psychiatric disorders were excluded from participation.

Sixteen patients (2 male, 14 female; age M = 24.88 years; SD = 7.85 years) meeting the DSM‐IV criteria for restrictive type AN according to the Structured Clinical Interview (SCID) were recruited from the inpatient service of the department of psychosomatic medicine and psychotherapy in Jena. Patients with a current comorbid Axis I or neurological disorder were excluded from this study. The mean age at onset of AN was 14.7 years (SD*  *=  3.6 years).

Descriptive sample characteristics are summarized in Table 1. Mann–Whitney *U*‐tests were calculated to determine if there were significant differences in education, body weight, eating pathology, state/trait anxiety, and overall impulsivity between healthy controls and AN patients. To account for the problem of multiple comparisons, we adjusted the statistical significance level using the false discovery rate (FDR) approach (Benjamini & Hochberg, 1995).

**TABLE 1:** Sample characteristics

	Healthy controls (*N* = 21)	Anorexia nervosa patients (*N* = 16)	*U* (Z; p_FDR_)
Education level (in years)	*M* = 11.91 (SD = 0.44)	*M* = 10.63 (SD = 1.41)	80.5 (−3.46; 0.0007)
Body‐mass‐index (BMI)	*M* = 24.3 (SD = 3.27)	*M* = 14.97 (SD = 1.41)	0 (−5.15; 0.0001)
Eating disorder inventory (EDI‐II short)	*M* = 159.53 (SD = 32.23)	*M* = 214.8 (SD = 34.26)	21 (−3.8; 0.0003)
State‐anxiety (STAI‐state)	*M* = 35.48 (SD = 8.54)	*M* = 47.94 (SD = 7.64)	50 (−3.62; 0.0004)
Trait‐anxiety (STAI‐trait)	*M* = 36.85 (SD = 6.64)	*M* = 52 (SD = 9.33)	50 (−4.1; 0.0001)
Overall impulsivity (BIS‐11)	*M* = 58.3 (SD = 5.47)	*M* = 61.19 (SD = 10.29)	121.5 (−1.23; 0.2196)

Abbreviations: f, female; FDR, false discovery rate; m, male; *M*, mean value; *N*, number of subjects; SD, standard deviation; *U*, indicates Mann–Whitney *U*‐tests.

All subjects were German native speakers, right‐handed according to the modified version of the Annetts Handedness Inventory (Briggs & Nebes, 1975) and provided written informed consent before participation. The Ethics Committee of the University of Jena approved the study protocol. All subjects were paid eight Euros per hour for their participation.

### 2.2 Experimental paradigm

The Go/Nogo paradigm is used to measure the ability to inhibit a pre‐potent response tendency. The Nogo‐signal, which triggers inhibitory processes, is presented unexpectedly following a Go‐signal, measuring the ability to inhibit an intended response. Our research group developed a modified version of the task previously published by Köhler et al. (2018). In short, at the beginning of the experiment, participants saw the word ‘READY’ in the middle of a screen. The ‘READY’ was replaced by a clay jug, in which water was dropping much frequented, representing our baseline measure. After varying time intervals, a stimulus appeared, which was either a Go or a Nogo trial. The Go stimuli are two kinds of transverse cracks, starting either from the left‐handed side or from the right‐handed side of the jug. The Nogo stimuli are two kinds of vertical cracks, starting either from the upper end or from the jug's bottom end. The stimuli were presented for 600 ms. The inter‐stimulus‐intervals (ISI) were 3800, 6000, and 8200 ms, which were presented one after the other and in equal parts, where the related stimulus‐onset asynchronies (SOAs) were 4400, 6600, and 8800 ms. Moreover, stimulus presentation onsets were jittered to prevent any signaling function of the water drops regarding the stimulus timing and to have a better time resolution of the hemodynamic responses. All subjects were asked to indicate which type of crack was presented by either pressing a button (with the right index finger) as fast as possible when a Go stimulus appeared or restraining their response when a Nogo stimulus appeared. Immediately after the stimulus presentation, water kept on dropping into the jug. There were more Go stimuli (in ∼74% of cases) than Nogo stimuli (in ∼26% of cases) to create a pre‐potent response tendency

In the present study, we adapted the experiment in total duration and length. Thus, the Go‐Nogo experiment now lasted for about 23 minutes, comprising 160 Go and 42 Nogo stimuli. Performance was assessed by the number of correct reactions in the Go and Nogo condition.

### 2.3 Assessment scales

Impulsivity was assessed by the Barratt Impulsivity Scale comprising attentional, motor and non‐planning subscales (BIS‐11; Patton et al., 1995). The purpose of the State‐Trait Anxiety Inventory (STAI; Laux et al., 1981) is to measure the presence and severity of current symptoms of anxiety as well as a general tendency to be anxious. Moreover, the Eating Disorder Inventory (EDI‐II, Garner, 1991) was used that evaluates symptoms and psychopathologic features of eating disorders. It consists of 91 items subdivided into 11 subscales.

### 2.4 MRI parameters

Data were collected on a 3T whole body‐system equipped with a 64‐element receive‐only head matrix coil. T2*‐weighted images were obtained using a gradient‐echo EPI sequence (TR =  2120 ms, TE =  36 ms, TA =  2100 ms, FOV = 224 mm^2^, acquisition matrix =  160 × 160 mm^2^, flip angle = 90°) with 104 interleaved transverse slices of 1.4 mm thickness, a multi‐band acceleration factor of 4 and with an in‐plane resolution of 1.4 × 1.4 mm^2^. A series of 606 whole‐brain volume sets were acquired in one session lasting approximately 23 minutes. High‐resolution anatomical T1‐weighted volume scans (MP‐RAGE) were obtained in sagittal orientation (TR = 2300 ms, TE = 3.03 ms, TI = 900 ms, flip angle = 9°, FOV = 256 mm × 256 mm, matrix 256 × 256, number of sagittal slices = 192, acceleration factor (PAT = 2) with an isotropic resolution of (1 × 1 × 1) mm^3^.

### 2.5 Physiological recordings during fMRI

During the fMRI scan, respiratory and cardiac signals were recorded simultaneously using an MR‐compatible BIOPAC MP150 polygraph (BIOPAC Systems Inc., Goleta, CA, USA) and digitized at 500 Hz. Respiratory activity was assessed by a strain gauge transducer incorporated in a belt tied around the chest, approximately at the level of the processus xiphoideus. The cardiac signal (photoplethysmograph (PPG) signal) was recorded using a pulse oximeter attached to the proximal phalanx of the index finger of the subject's left hand.

To remove MRI‐related or movement artifacts, the PPG signal was band‐pass filtered (0.05−3 Hz), and the respiratory signal was low‐pass filtered with a cutoff frequency of 10 Hz. Pulse‐wave onsets were automatically extracted by detecting peaks of the temporal derivative of the filtered PPG signal (Schumann et al., 2018). The quality of peak detection was visually inspected by an expert and corrected when necessary. SC was measured continuously (constant voltage technique) at the left hands’ palm with Ag/AgCl electrodes placed at the thenar and hypothenar eminence. All signals were sampled at 500 Hz and amplified in a frequency range of 0.05−3 Hz for PPG, 0.05–10 Hz for respiration and 0–10 Hz for SC. Respiration and SC were offline median filtered (window of 250 samples) and smoothed (over 500 samples) to reduce MRI‐related artifacts.

### 2.6 fMRI preprocessing

Data analysis was performed using SPM12 (https://www.fil.ion.ucl.ac.uk/spm) and AFNI software package (https://afni.nimh.nih.gov/). The first four images were discarded to ensure a steady‐state tissue magnetization condition. Time‐locked cardiac and respiratory artifacts, as well as slow blood oxygenation level fluctuations, were removed using RETROICOR (Glover et al., 2000) and respiration volumes per time regressors (Birn et al., 2008). RETROICOR and RVT regressors were generated on a slice‐wise basis by AFNI's “RetroTS.m” script (Jo et al., 2010).

Further preprocessing steps of the fMRI data included slice timing correction, rigid body realignment to the mean of all images, and functional and anatomical data alignment. Afterwards, images were normalized to the MNI space using the DARTEL procedure integrated into SPM12 (Ashburner, 2007) and smoothed with a Gaussian kernel of 6 mm full‐width at half‐maximum.

### 2.7 Physiological data analysis

Event‐related skin conductance (SC) responses (SCR) were estimated on SC signals after removing the slowly varying component (SC smoothed over 10.000 samples). SC time courses at the onset of each Go/Nogo stimulus (reference time *t*  =  0, lasting 6 s) were extracted and normalized to baseline (mean SC in 1 s before stimulus‐onset). We averaged responses per subject in C and IC Nogo trials. Reactions of SC were quantified in terms of area under the curve by integrating subject‐specific responses (Bach et al., 2010). SCR is given in normalized units (n.u.) and was compared between trials using the Wilcoxon signed‐rank test (IC vs. C).

### 2.8 fMRI data analysis

An ANOVA design with the within‐subject factor task (correct Go, correct Nogo, incorrect Nogo) and the between‐subject factor group (controls, patients) was performed.

Using the SPM12 software, a fixed‐effects model with an event‐related design including correct Go, correct Nogo as well as incorrect Nogo responses at the single‐subject level was built to create contrast images of parameter estimates. The remaining stimuli, i.e. misses on Go trials, as well as the individual movement parameters, were also entered in the single‐subject fixed‐effects model as covariates of no interest. Further, one additional regressor of no interest was included to modulate response speed in the Go condition to control reaction time and improve the model's accuracy.

Final contrast estimates were entered into a second‐level analysis. At the second level, a random‐effects full‐factorial design was used to investigate neuronal activation in the groups (i.e. patients and controls) and conditions (Go, C Nogo and IC Nogo). To get insight in error processing, a one‐way ANOVA with group (patients vs. controls) as the between‐subjects factor was performed for the C Nogo and IC Nogo condition. Thus, we compared unsuccessful response inhibition (IC Nogo) with successful response inhibition (C Nogo) within and between groups. The statistical comparisons were thresholded on the voxel‐level at *p*  <  0.005 (uncorrected) and *p*  <  0.05 FWE corrected at the cluster‐level.

For correlational analysis, a regression model was built at the second level, including activation maps in the error processing contrast (IC Nogo > C Nogo). Individual performance and averaged increases SCR (IC Nogo – C Nogo) in patients and controls.

If not indicated, within‐ and between‐group analyses were based on a voxel‐based threshold of 0.005 (uncorrected) and were false discovery rate (FDR; Benjamini & Hochberg, 1995) cluster corrected.

### 2.9 Behavioral data analysis

Behavioral data analyses were performed using SPSS Statistics V24 (IBM Corp., Released 2016). The non‐parametric Mann–Whitney *U*‐test was used to test for between‐group differences in performance. Within group, differences were analyzed using the Wilcoxon signed‐rank test. To account for the problem of multiple comparisons, we adjusted the statistical significance level using the FDR approach (Benjamini & Hochberg, 1995).

## 3 RESULTS

### 3.1 Task performance

There were no statistically significant differences in Go accuracy, Go response times or Nogo accuracy (Table 2). Within groups there were significantly slower responses after error commission than after pre‐error responses (HC δ = 420.46 ms, SD = 415.2 ms, Z = −3.53, *p* < 0.001; AN δ = 486.88 ms, SD = 413.26 ms, Wilcoxon signed‐rank test: Z = −3.15, *p* = 0.002). However, there was no significant difference between groups in post‐error slowing.

**TABLE 2:** Behavioral performance

	Healthy controls (*N* = 21)	Anorexia nervosa patients (*N* = 16)	*U* (Z, p_FDR_)
Go accuracy	*M* = 159.38 (SD = 1.16)	*M* = 157.5 (SD = 2.81)	97.5 (−2.39, 0.068)
Nogo accuracy	*M* = 34.38 (SD = 4.46)	*M* = 33.25 (SD = 4.54)	144.5 (−0.72, 0.5)
Go response time (in ms)	*M* = 475.3 (SD = 85.19)	*M* = 490.54 (SD = 36.16)	100 (−2.09, 0.074)
Post‐error slowing (in ms)	*M* = 420.46 (SD = 415.21)	*M* = 486.88 (SD = 413.24)	146 (−0.67, 0.5)

Abbreviations: FDR, false discovery rate; *M*, mean value; ms, milliseconds; *N*, number of subjects; SD, standard deviation; *U*, indicates Mann–Whitney *U*‐tests.

### 3.2 Indices of the peripheral autonomic nervous system

**FIGURE 1:**
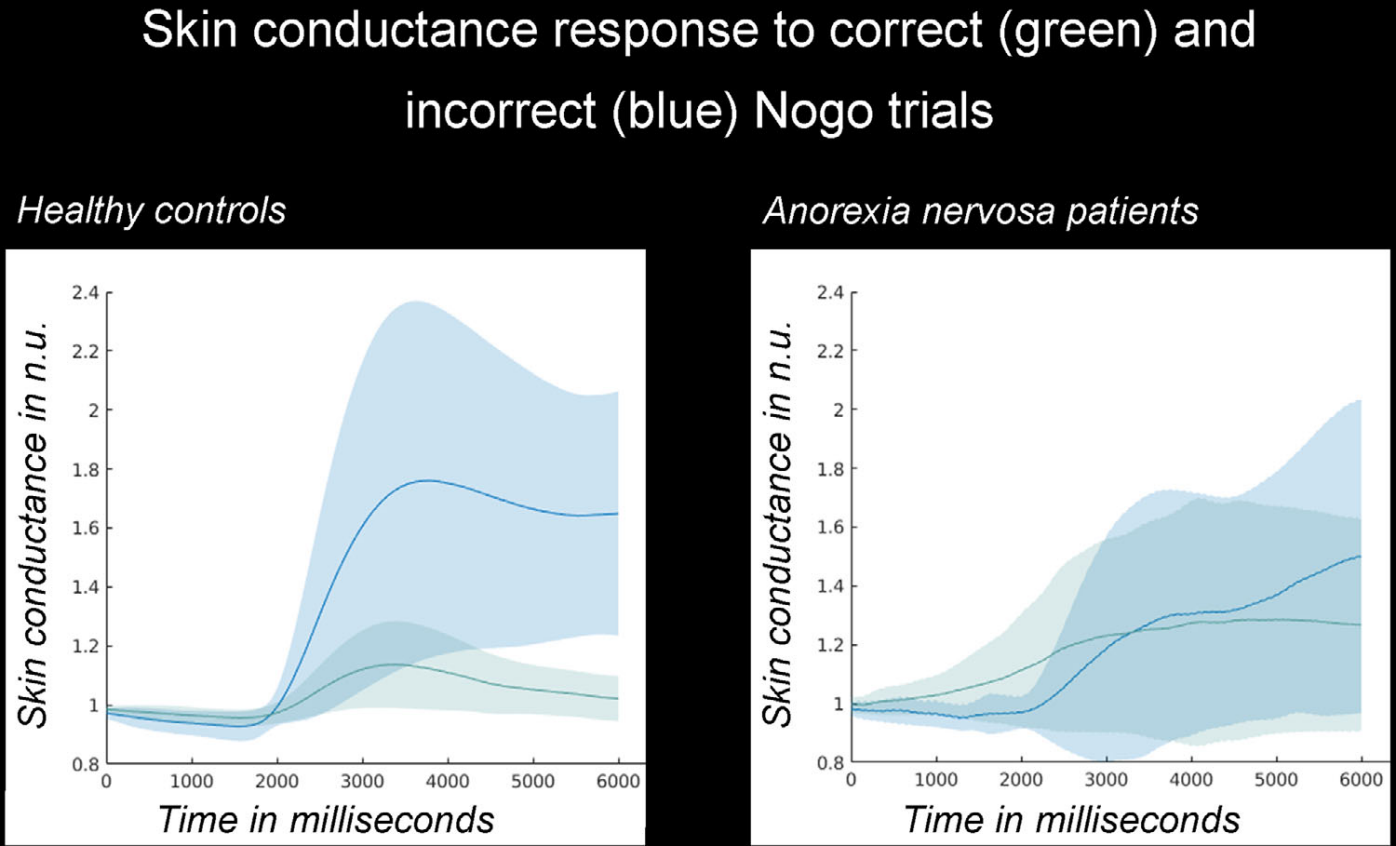
FIGURE 1 Linking physiological indices to behavioral performance: Mean skin conductance responses in the correct Nogo (green) and incorrect Nogo (blue) trials are shown (areas under the curve indicate standard deviations). Abbreviations: n. u., normalized units; SC, skin conductance.

No difference was observed in skin conductance between groups (Table 3). However, SCR during correct Nogo trials (C Nogo), during incorrect Nogo trials (IC Nogo) as well as their difference were significantly lower in AN patients. In Figure 1, averaged SCR are depicted for correct and incorrect Nogo trials, normalized to baseline. In HC, SCR were significantly higher in incorrect compared to correct trials (Z = −3.77, *p* < 0.001), but not in AN patients (Z = 0, *p* = n.s.).

**TABLE 3:** Indices of the peripheral autonomic nervous system

	Healthy controls (*N* = 21)	Anorexia nervosa patients (*N* = 16)	Group difference *U* (Z, p_FDR_)
Skin conductance level in μS	*M* = 0.95 (SD = 0.7)	*M* = 1.23 (SD = 1.36)	163 (−0.15, 0.878)
Skin conductance responses (C Nogo) in n.u.	*M* = 3088.22 (SD = 153.81)	*M* = 3576.23 (SD = 797.98)	88 (−2.45, 0.021)
Skin conductance responses (IC Nogo) in n.u.	*M* = 4050.1 (SD = 916.84)	*M* = 3387.59 (SD = 499.1)	86 (−2.51, 0.021)
Skin conductance responses (IC Nogo vs. C Nogo) in n.u.	*M* = 961.85 (SD = 897.1)	*M* = −188.65 (SD = 1096.24)	75 (−2.85, 0.015)

Abbreviations: C, correct Nogo; FDR, false discovery rate; IC, incorrect Nogo; *M*, mean value; *N*, number of subjects; n.u., normalized units; SD, standard deviation; *U*, indicates Mann–Whitney *U*‐tests.

### 3.3 Analyses of neuronal response patterns

**FIGURE 2:**
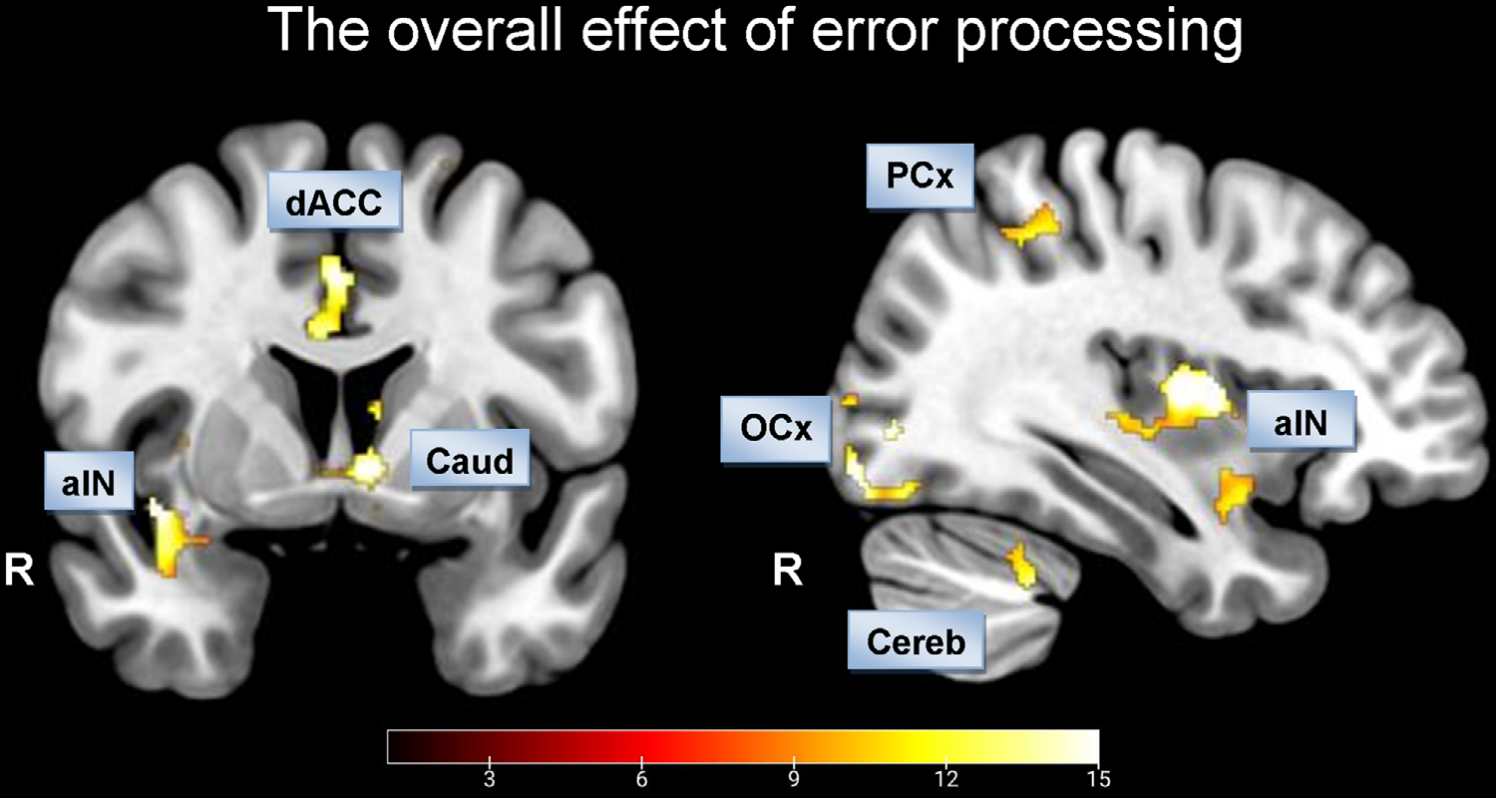
FIGURE 2 Main effect of group: voxel‐level *p* < 0.005 uncorrected, cluster‐level *p* < 0.05, FDR corrected. Abbreviations: aIN, anterior insula; Caud, nucleus caudatus; Cereb, Cerebellum; dACC, dorsal anterior cingulate cortex; OCx, occipital cortex; PCx, parietal cortex; R, right.

To test for overall group differences, we performed an analysis of variance without proposing effect directions. In this analysis, we found differences in neuronal activations in key regions comprising the salience network, e.g., the ACC, and aIN (Figure 2, Table S1; voxel‐level: *p*  <  0.005 uncorrected; cluster‐level *p*  <  0.05, FDR corrected).

We then used post‐hoc t‐tests to analyze neuronal activation patterns in response to errors. More precisely, to examine error processing, we compared mistakenly answered (incorrect (IC) Nogo) to correctly inhibited (correct (C) Nogo) trials of the Go/Nogo paradigm.

**FIGURE 3:**
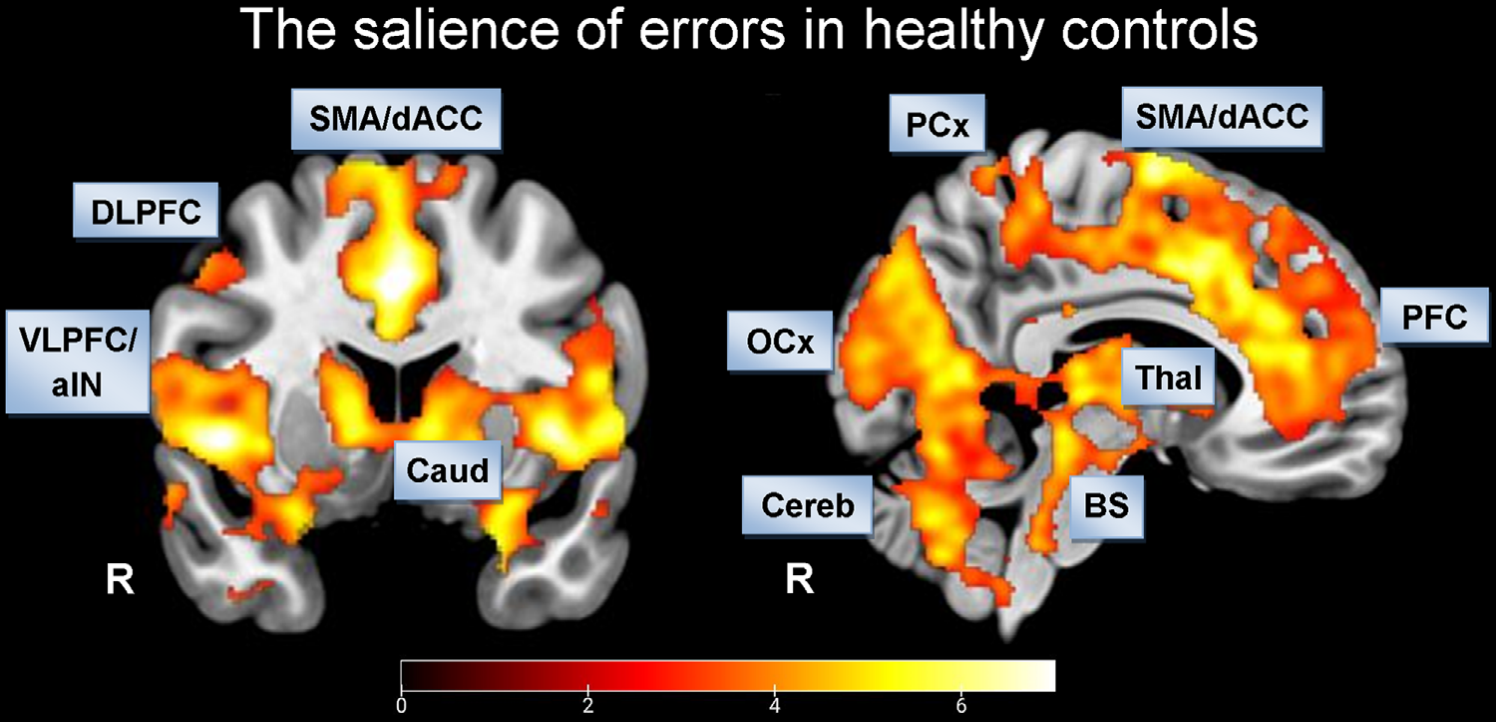
**FIGURE 3** Error processing in healthy controls: voxel‐level *p* < 0.001 uncorrected, cluster‐level *p* < 0.05, FDR corrected. Abbreviations: aIN, anterior insula; Caud, nucleus caudatus; Cereb, Cerebellum; dACC, dorsal anterior cingulate cortex; DLPFC, dorsolateral prefrontal cortex; OCx, occipital cortex; PCx, parietal cortex; PFC, prefrontal cortex; R, right; SMA, supplementary motor area; VLPFC, ventrolateral prefrontal cortex.

We found increased BOLD activations, especially in medial and lateral prefrontal regions, amygdala, hippocampus, dACC, aIN, and occipital cortex, in response to errors in HC (Figure 3; Table 4).

**TABLE 4:** Response to errors in healthy controls

				MNI coordinates			
Region of activation	Right/left	Brodmann area	Cluster size	x	y	z	T value
Precentral gyrus	R	4	124997	45	−13	40	8.76
Anterior cingulate gyrus	R	32		2	11	40	8.47
Supplementary motor area	R	6		1	10	39	8.45
Cingulate gyrus	L	24		−3	3.5	41	8.26
Precuneus	L	31		−5	−42	53	6.74
Caudate nucleus	R			17	17	−3	6.46
Anterior insula	L	13		−32	26	5	6.10
Thalamus	L			0	−7	7	5.97
Brainstem	L			−3	−28	−19	5.94
Anterior insula	R	13		35	26	2	5.67
Occipital gyrus	L	17		−5	−85	17	5.37
Thalamus	R			9	−13	2	5.29
Caudate nucleus	L			−2	13	1	4.73
Middle frontal gyrus	L	9		−26	41	40	3.67
Cerebellum	R		364	23	−45	−49	4.41

*Note*: Maxima of regions showing significant BOLD activation differences when comparing incorrect and correct Nogo trials (error processing contrast) in healthy controls at the whole‐brain level (voxel‐level *p* < 0.001 uncorr., cluster‐level, *p* < 0.05, FDR corr.).

Abbreviations: L, left; M, middle; R, right.

**FIGURE 4:**
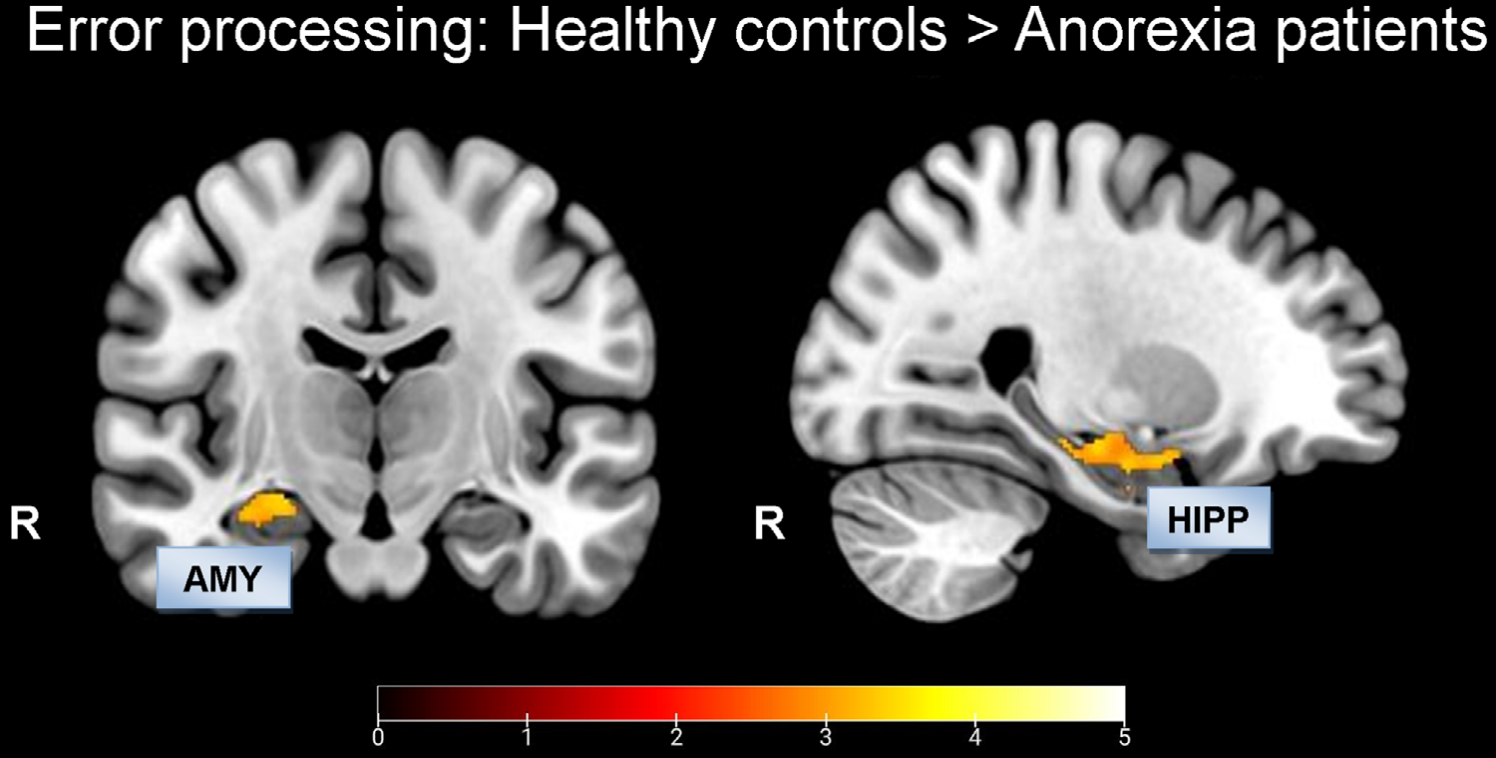
FIGURE 4 Between‐group comparison in the error processing contrast: voxel‐level *p* < 0.005 uncorrected, cluster‐level *p* < 0.05, FDR corrected. Abbreviations: AMY, amygdala; AN, anorexia nervosa patients; C, correct; HC, healthy controls; HIPP, hippocampus; IC, incorrect; R, right.

In contrast, there were no significant BOLD activations during error processing in AN patients. Particularly interesting, however, is that the between‐group t‐contrast revealed significantly increased neuronal responses in the amygdala (*x* = 26, *y* = 1, *z* = −18, *t* = 3.38) and hippocampus (*x* = 24, *y* = −16, *z* = −15, *t* = 3.54) in HC compared to patients (Figure 4).

### 3.4 Relations between performance, core regions of the salience network and skin conductance indices

**FIGURE 5:**
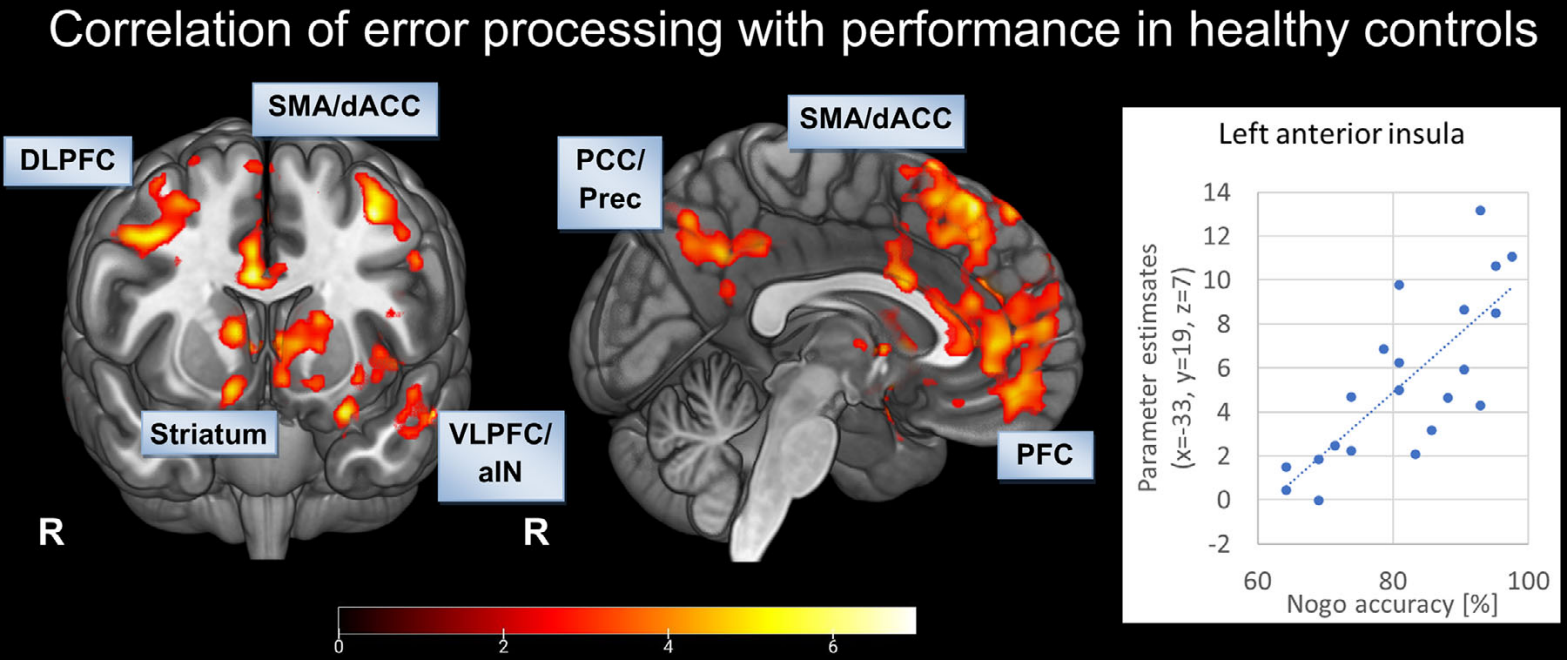
FIGURE 5 Correlation of activations during error processing and task performance in healthy controls: voxel‐level *p* < 0.005 uncorrected, cluster‐level *p* < 0.05, FDR corrected. Abbreviations: aIN, anterior insula; dACC, dorsal anterior cingulate cortex; DLPFC, dorsolateral prefrontal cortex; PFC, prefrontal cortex; PCC, posterior cingulate cortex; Prec, precuneus; R, right; SMA, supplementary motor area; VLPFC, ventrolateral prefrontal cortex.

We investigated response behavior associated with neuronal activation patterns during the task and SCR in healthy controls and AN patients. The regression analysis revealed a pattern of significant clusters correlated with achieved performance, including the anterior insula, cingulate cortex, and amygdala (Figure 5). In addition, striatal, as well as medial and lateral prefrontal regions, showed activation proportional to performance (Table 5). At this statistical level, there were no significant associations to SCR in controls. In patients, there were no correlations with either performance or to SCR.

**TABLE 5:** Correlation of activations during error processing and task performance in healthy controls

				MNI coordinates			
Region of activation	Right/left	Brodmann area	Cluster size	*x*	*y*	*z*	T value
Lateral frontal gyrus	L	6	2117	−36	8	49	8.55
Middle temporal gyrus	L	21	2426	−63	−33	−9	7.39
Putamen	L		21680	−20	16	−1	6.76
Middle frontal gyrus	R/L	8		3	35	52	6.71
Cingulate gyrus	R/L	24		5	10	28	6.66
Lateral frontal gyrus	R	45	805	56	34	5	6.08
Angular gyrus	L	40	1286	−53	−52	31	6.06
Nucleus accumbens	R	25	562	12	10	−13	5.36
Amygdala	R			21	−7	−12	4.97
Precuneus	L/R	7	1600	0	−66	47	5.09
Middle temporal gyrus	R	21	1235	50	−34	−3	5.05

*Note*: Correlation of activations during error processing and task performance in healthy controls at the whole‐brain level (voxel‐level *p* < 0.005 uncorr., cluster‐level, *p* < 0.05, FDR corr.); Abbreviations: L, left; R, right.

## 4 DISCUSSION

In the present study, we investigated response inhibition in acute AN patients. In the Go/Nogo task, patients and healthy controls performed equally well. Healthy subjects elicited stronger SCR after failed Nogo trials indicating sympathetic arousal on errors. In contrast, patients did not differ in SCR between correct and incorrect Nogo trials. Accordingly, we observed diminished neuronal responses during error processing in the amygdala and hippocampus in patients compared to controls. Finally, we found significant positive correlations between Nogo performance and activations of core regions of the salience network during errors in controls but not in patients. Results of this study indicate disturbed error processing in AN patients.

### 4.1 Error processing in AN

When a person is presented with a stimulus, they must decide whether a response is required and which response is appropriate. Proactive inhibitory control encompasses response withholding or behavioral adjustments when the need for a response is uncertain (Bartholdy et al., 2016). Thus, it is probable that the degree to which proactive inhibition is applied is related to how much a person tolerates uncertainty. Thus the perfectionism and intolerance of uncertainty in AN (Brown et al., 2017), the decision making of AN patients is likely dominated by proactive inhibition. For instance, Bartholdy et al. (2017) investigated whether proactive and reactive inhibitory control differed across eating disorders. Whereas there were no group differences in reactive inhibitory, AN patients showed greater proactive inhibition than HC.

In healthy (reactive) controls, adaption of behavior after error indicates cognitive flexibility. Thus, adequate error processing is essential to good performance and cognitive flexibility. However, patients do not show clearer neuronal activation on error trials than correct trials as do control subjects. Accordingly, in the group comparison regarding the error processing contrast, we found significantly increased neuronal responses in the amygdala and hippocampus in HC compared to patients. This result is in line with the finding of Bang et al. (2016), who investigated AN patients using fMRI and an emotional conflict task and found that patients had fewer BOLD responses than controls in the amygdala and hippocampus during the processing of salient stimuli. In general, limbic brain structures are the neuronal basis of emotions and include the amygdala and hippocampus (Yang & Wang, 2017). Thus, in regard to our findings, we assume that AN patients compared to HC show enhanced cognitive control during the whole response control task but show less emotional responses to salient, i.e., erroneous, stimuli.

Moreover, correlational analyses revealed that healthy participants who showed stronger neuronal reactions to errors in the salience network performed better in the task. Thus, successful participants adjusted their behavior according to their response failures. In patients, performance was not dependent on neuronal error processing. Hence, correlational findings align with the identified neuronal and autonomic response patterns, where no relation between performance, core regions of the salience network and skin conductance indices were found in AN patients.

### 4.2 Role of the salience network in error processing

Errors are salient stimuli that lead to activation of the salience network. This network initiates switches between other functional neuronal networks facilitating access to memory resources and attention. The insula seems to be involved especially in the detection of salient stimuli, whereas the dACC induces the preparation of motor readiness (Menon, 2011). Switching between functional networks is crucial for cognitive flexibility (Pedersen et al., 2018).

In a review by Steward et al. (2018), the maintenance of restricted eating behavior in AN patients was associated with increased neuronal activations in the executive control network. Executive control becomes relevant in, for instance, response inhibition and performance monitoring (Braver & Ruge, 2006). The persistent hyper‐activation of the executive control network seems to be the central correlate of the overregulated and inflexible response control in anorexia patients.

### 4.3 Physiological responses to errors

The present study results extend the finding of attenuated responses to errors in AN to include autonomic physiological reactions. We found that SCR in failed Nogo‐trials were lower in patients than in controls. The fact that patients showed increased SCR in correct Nogo trials compared to controls supported our impression that patients engaged in the task with motivation. However, failed trials did not increase SCR, indicating a damped affective sympathetic reaction to behavioral errors.

That patients are physiologically able to elicit normal SCR has been demonstrated in previous studies. For instance, orienting restrictive type patient responses to loud tones are similar to controls (Calloway et al., 1983). Significantly stronger SCR occur to food‐related stimuli (Léonard et, al.,1998; Gorini et al., 2010). Analyses of skin conductance during cognitive processing are rare. Tchanturia et al. (2007) found that losses in a gambling task induced attenuated SCR in AN compared to controls. Nandrino et al. (2012) found that AN patients made more errors categorizing emotional pictures, especially for neural content that were not associated with SCR increases. Furthermore, the authors reported a dissociation of SCR and the cognitive evaluation of emotional pictures and concluded that the sensitivity to emotional stimuli is modulated by controlled processes (Soussignan et al., 2010).

While AN patients had normal electrodermal responses for positive cues, their subjective ratings and the number of classification errors were higher than for the control participants. These results support the idea that AN patients have difficulty evaluating positive stimulus intensity and that they present a dissociation between objective and subjective measures of hedonic processes. These findings are partially in accordance with Miller et al. (2003), who observed that AN patients lacked a relationship with affective self‐appraisal, unlike the control participants. Such a dissociation was also observed by Soussignan et al. (2010) using visual and olfactory cues. The authors show that, although the automatic processing of pleasantness is altered in anorexia, sensitivity to pleasant stimuli is modulated by controlled processes. It can thus be hypothesized that, as AN patients identify the pleasant stimuli less frequently, they are likely to over‐estimate the intensity of pleasant stimuli when seeking to adjust to social learning processes or expectations. This evaluation would correspond to controlled processes that are not related to physiological responses.

Moreover, correlational analyses revealed that healthy participants, who showed stronger neuronal reactions in the salience network to errors, performed better in the task. Thus, successful participants adjusted their behavior according to their response failures. In patients, performance was not dependent on neuronal error processing. Hence, the correlational findings are in line with the identified neuronal and autonomic response patterns where no relations between performance, core regions of the salience network and skin conductance indices were found in AN patients.

In conclusion, we propose that peripheral response behavior related to error processing and the associated neuronal processes are more inflexible and detached from each other in AN patients. This is very likely to be of consequence as patients seem to be over‐regulated at behavioral and neuronal levels, with an accompanying rigid peripheral autonomic nervous system.

### 4.4 Limitations and outlook

One limitation of our study is the small sample size. In consequence, the findings should be generalized with care. Furthermore, we did not analyze the reactions of other parts of the autonomic nervous system, e.g. cardiac or pupillary responses. Thus, these should be included in future studies.

Our study aimed to shed further light on neuronal and autonomic responses during error processing in patients suffering from AN. With our research, we made an essential contribution to current research regarding error processing in this fatal disease. It seems that AN patients lack neuronal and autonomic responses to errors that might impede flexible adaption of behavior. In future studies, we want to test whether cognitive rigidity is able to predict the probability of remission from AN. Understanding the neuro‐cognitive underpinnings of affective dysregulation in the brain of patients with AN might even also guide the development of additional treatment strategies. For instance, biofeedback of skin conductance is able to enhance sympathetic arousal by modulating brain activity, especially in the PF (Nagai et al., 2004). Therefore, biofeedback‐assisted training to improve emotional skills might be a useful therapeutic add‐on and a promising disease prevention strategy.

## Supporting information

Suporting information

## Data Availability

The data that support the findings of this study are available from the corresponding author upon reasonable request.

## References

[brb32235-bib-0001] Arcelus, J., Mitchell, A. J., Wales, J., & Nielsen, S. (2011). Mortality rates in patients with anorexia nervosa and other eating disorders: A meta‐analysis of 36 studies. Archives of General Psychiatry, 68(7), 724–731. doi: 10.1001/archgenpsychiatry.2011.74 21727255

[brb32235-bib-0002] Ashburner, J. (2007). A fast diffeomorphic image registration algorithm. Neuroimage, 38(1), 95–113. 10.1016/j.neuroimage.2007.07.007 17761438

[brb32235-bib-0003] Bach, D. R., Friston, K. J., & Dolan, R. J. (2010). Analytic measures for quantification of arousal from spontaneous skin conductance fluctuations. International Journal of Psychophysiology, 76(1), 52–55. 10.1016/j.ijpsycho.2010.01.011 20144665 PMC2877802

[brb32235-bib-0004] Bang, L., Rø, Ø., & Endestad, T. (2016). Amygdala alterations during an emotional conflict task in women recovered from anorexia nervosa. Psychiatry Research: Neuroimaging, 248, 126–133. 10.1016/j.pscychresns.2015.12.008 26778366

[brb32235-bib-0005] Bartholdy, S., Campbell, I. C., Schmidt, U., & O'Daly, O. G. (2016). Proactive inhibition: An element of inhibitory control in eating disorders. Neuroscience and Biobehavioral Reviews, 71, 1–6. 10.1016/j.neubiorev.2016.08.022 27565516

[brb32235-bib-0006] Bartholdy, S., Rennalls, S. J., Jacques, C., Danby, H., Campbell, I. C., Schmidt, U., & O'Daly, O. G. (2017). Proactive and reactive inhibitory control in eating disorders. Psychiatry Research, 255, 432–440. 10.1016/j.psychres.2017.06.073 28672226 PMC5555256

[brb32235-bib-0007] Benjamini, Y., & Hochberg, Y. (1995). Controlling the false discovery rate: A practical and powerful approach to multiple testing. Journal of the Royal Statistical Society: Series B (Methodological), 57(1), 289–300. 10.1111/j.2517-6161.1995.tb02031.x

[brb32235-bib-0008] Birn, R. M., Smith, M. A., Jones, T. B., & Bandettini, P. A. (2008). The respiration response function: The temporal dynamics of fMRI signal fluctuations related to changes in respiration. Neuroimage, 40(2), 644–654. 10.1016/j.neuroimage.2007.11.059 18234517 PMC2533266

[brb32235-bib-0009] Braver, T. S., & Ruge, H. (2006). Functional neuroimaging of executive functions. MIT Press.

[brb32235-bib-0010] Briggs, G. G., & Nebes, R. D. (1975). Patterns of hand preference in a student population. Cortex; A Journal Devoted to the Study of the Nervous System and Behavior, 11(3), 230–238. 10.1016/s0010-9452(75)80005-0 1204363

[brb32235-bib-0011] Brown, M., Robinson, L., Campione, G. C., Wuensch, K., Hildebrandt, T., & Micali, N. (2017). Intolerance of uncertainty in eating disorders: A systematic review and meta‐analysis. European Eating Disorders Review, 25(5), 329–343. 10.1002/erv.2523 28544668

[brb32235-bib-0012] Buzzichelli, S., Marzola, E., Amianto, F., Fassino, S., & Abbate‐Daga, G. (2018). Perfectionism and cognitive rigidity in anorexia nervosa: Is there an association? European Eating Disorders Review, 26(4), 360–366. 10.1002/erv.2591 29635827

[brb32235-bib-0013] Calloway, P., Fonagy, P., & Wakeling, A. (1983). Autonomic arousal in eating disorders: Further evidence for the clinical subdivision of anorexia nervosa. British Journal of Psychiatry, 142, 38–42. 10.1192/bjp.142.1.38. 6572539

[brb32235-bib-0014] Damasio, A. R. (2003). Looking for Spinoza: Joy, sorrow, and the feeling brain. Harcourt Brace & Co.

[brb32235-bib-0015] Garner, D. M. (1991). Eating disorder inventory 2. Professional manual. Psychological Assessment Resources.

[brb32235-bib-0016] Geisler, D., Ritschel, F., King, J. A., Bernardoni, F., Seidel, M., Boehm, I., Runge, F., Goschke, T., Roessner, V., Smolka, M. N., & Ehrlich, S. (2017). Increased anterior cingulate cortex response precedes behavioural adaptation in anorexia nervosa. Scientific Reports, 7, 42066. 10.1038/srep42066 28198813 PMC5304157

[brb32235-bib-0017] Glover, G. H., Li, T. Q., & Ress, D. (2000). Image‐based method for retrospective correction of physiological motion effects in fMRI: RETROICOR. Magnetic Resonance in Medicine, 44(1), 162–167. 10.1002/1522-2594(200007) 44:1<162::aid‐mrm23>3.0.co;2‐e 10893535

[brb32235-bib-0018] Gorini, A., Griez, E., Petrova, A., & Riva, G. (2010). Assessment of the emotional responses produced by exposure to real food, virtual food and photographs of food in patients affected by eating disorders. Annals of General Psychiatry, 9, 30. 10.1186/1744-859X-9-30. 20602749 PMC2914081

[brb32235-bib-0019] Goulden, N., Khusnulina, A., Davis, N. J., Bracewell, R. M., Bokde, A. L., McNulty, J. P., & Mullins, P. G. (2014). The salience network is responsible for switching between the default mode network and the central executive network: Replication from DCM. Neuroimage, 99, 180–190. 10.1016/j.neuroimage.2014.05.052 24862074

[brb32235-bib-0020] IBM Corp. (Released 2016).

[brb32235-bib-0021] Jagielska, G., & Kacperska, I. (2017). Outcome, comorbidity and prognosis in anorexia nervosa. Psychiatria Polska, 51(2), 205–218. 10.12740/pp/64580 28581532

[brb32235-bib-0022] Jo, H. J., Saad, Z. S., Simmons, W. K., Milbury, L. A., & Cox, R. W. (2010). Mapping sources of correlation in resting state FMRI, with artifact detection and removal. Neuroimage, 52(2), 571–582. 10.1016/j.neuroimage.2010.04.246 20420926 PMC2897154

[brb32235-bib-0023] Kerr, K. L., Moseman, S. E., Avery, J. A., Bodurka, J., Zucker, N. L., & Simmons, W. K. (2016). Altered insula activity during visceral interoception in weight‐restored patients with anorexia nervosa. Neuropsychopharmacology: Official Publication of the American College of Neuropsychopharmacology, 41(2), 521–528. 10.1038/npp.2015.174 26084229 PMC5130127

[brb32235-bib-0024] Köhler, S., Schumann, A., de la Cruz, F., Wagner, G., & Bär, K. J. (2018). Towards response success prediction: An integrative approach using high‐resolution fMRI and autonomic indices. Neuropsychologia, 119, 182–190. 10.1016/j.neuropsychologia.2018.08.003 30092240

[brb32235-bib-0025] Laux, L., Glanzmann, P., Schaffner, P., & Spielberger, C. D. (1981). Das State‐Trait‐Angstinventar. Beltz Test.

[brb32235-bib-0026] Léonard, T., Pepinà, C., Bond, A., & Treasure, J. (1998), Assessment of test‐meal induced autonomic arousal in anorexic, bulimic and control females. European Eating Disorders Review, 6, 188–200. 10.1002/(SICI)1099-0968(199809) 6:3<188::AID‐ERV227 >3.0.CO; 2‐G

[brb32235-bib-0027] Menon, V. (2011). Large‐scale brain networks and psychopathology: A unifying triple network model. Trends in Cognitive Sciences, 15(10), 483–506. 10.1016/j.tics.2011.08.003 21908230

[brb32235-bib-0028] Miller, S. P., Redlich, A. D., & Steiner, H. (2003). The stress response in anorexia nervosa. Child Psychiatry and Human Development, 33(4), 295–306. 10.1023/a:1023036329399. 12723902

[brb32235-bib-0029] Nagai, Y., Critchley, H. D., Featherstone, E., Trimble, M. R., & Dolan, R. J. (2004). Activity in ventromedial prefrontal cortex covaries with sympathetic skin conductance level: A physiological account of a "default mode" of brain function. Neuroimage, 22(1), 243–251. 10.1016/j.neuroimage.2004.01.019. 15110014

[brb32235-bib-0030] Nandrino, J. L., Berna, G., Hot, P., Dodin, V., Latrée, J., Decharles, S., & Sequeira, H. (2012). Cognitive and physiological dissociations in response to emotional pictures in patients with anorexia. Journal of Psychosomatic Research, 72(1), 58–64. 10.1016/j.jpsychores.2011.11.003. Epub 2011 Nov 29. PMID: 22200524. 22200524

[brb32235-bib-0031] Oberndorfer, T. A., Kaye, W. H., Simmons, A. N., Strigo, I. A., & Matthews, S. C. (2011). Demand‐specific alteration of medial prefrontal cortex response during an inhibition task in recovered anorexic women. International Journal of Eating Disorders, 44(1), 1–8. 10.1002/eat.20750 20127942

[brb32235-bib-0032] Patton, J. H., Stanford, M. S., & Barratt, E. S. (1995). Factor structure of the Barratt impulsiveness scale. Journal of Clinical Psychology, 51(6), 768–774. 10.1002/1097-4679(199511)51:6<768::aid‐jclp2270510607>3.0.co;2‐1 8778124

[brb32235-bib-0033] Pedersen, M., Zalesky, A., Omidvarnia, A., & Jackson, G. D. (2018). Multilayer network switching rate predicts brain performance. Proceedings of the National Academy of Sciences of the United States of America, 115(52), 13376–13381. 10.1073/pnas.1814785115 30545918 PMC6310789

[brb32235-bib-0034] Phillips, M. L., Drevets, W. C., Rauch, S. L., & Lane, R. (2003). Neurobiology of emotion perception II: Implications for major psychiatric disorders. Biological Psychiatry, 54(5), 515–528. 10.1016/s0006-3223(03)00171-9 12946880

[brb32235-bib-0035] Pieters, G. L. M., de Bruijn, E. R. A., Maas, Y., Hulstijn, W., Vandereycken, W., Peuskens, J., & Sabbe, B. G. (2007). Action monitoring and perfectionism in anorexia nervosa. Brain and Cognition, 63(1), 42–50. 10.1016/j.bandc.2006.07.009 16962223

[brb32235-bib-0036] Schachter, S., & Singer, J. E. (1962). Cognitive, social, and physiological determinants of emotional state. Psychological Review, 69, 379–399. 10.1037/h0046234. 14497895

[brb32235-bib-0037] Schumann, A., Köhler, S., de la Cruz, F., Güllmar, D., Reichenbach, J. R., Wagner, G., & Bär, K. J. (2018). The use of physiological signals in brainstem/midbrain fMRI. Frontiers in Neuroscience, 12, 718. 10.3389/fnins.2018.00718 30386203 PMC6198067

[brb32235-bib-0038] Seeley, W. W., Menon, V., Schatzberg, A. F., Keller, J., Glover, G. H., Kenna, H., Reiss, A. L., & Greicius, M. D. (2007). Dissociable intrinsic connectivity networks for salience processing and executive control. Journal of Neuroscience, 27(9), 2349–2356. 10.1523/jneurosci.5587-06.2007 17329432 PMC2680293

[brb32235-bib-0039] Soussignan, R., Schaal, B., Rigaud, D., Royet, J. P., & Jiang, T. (2010). Hedonic reactivity to visual and olfactory cues: Rapid facial electromyographic reactions are altered in anorexia nervosa. Biological Psychology, 86(3), 265–272. 10.1016/j.biopsycho.2010.12.007. 21185351

[brb32235-bib-0040] Steward, T., Menchon, J. M., Jimenez‐Murcia, S., Soriano‐Mas, C., & Fernandez‐Aranda, F. (2018). Neural network alterations across eating disorders: A narrative review of fMRI studies. Current Neuropharmacology, 16(8), 1150–1163. 10.2174/1570159X15666171017111532 29046154 PMC6187750

[brb32235-bib-0041] Tchanturia, K., Liao, P. C., Uher, R., Lawrence, N., Treasure, J., & Campbell, I. C. (2007). An investigation of decision making in anorexia nervosa using the Iowa Gambling Task and skin conductance measurements. Journal of the International Neuropsychological Society, 13(4), 635–641. 10.1017/s1355617707070798 17521482

[brb32235-bib-0042] Treasure, J., Zipfel, S., Micali, N., Wade, T., Stice, E., Claudino, A., Schmidt, U., Frank, G. K., Bulik, C. M., & Wentz, E. (2015). Anorexia nervosa. Nature Reviews Disease Primers, 1, 15074. 10.1038/nrdp.2015.74 27189821

[brb32235-bib-0043] Wierenga, C., Bischoff‐Grethe, A., Melrose, A. J., Grenesko‐Stevens, E., Irvine, Z., Wagner, A., Schmidt, U., Frank, G. K., Bulik, C. K., & Kaye, W. H. (2014). Altered BOLD response during inhibitory and error processing in adolescents with anorexia nervosa. PLOS One, 9(3), e92017. 10.1371/journal.pone.0092017 24651705 PMC3961291

[brb32235-bib-0044] Yang, Y., & Wang, J.‐Z. (2017). From structure to behavior in basolateral amygdala‐hippocampus circuits. Frontiers in Neural Circuits, 11(86). 10.3389/fncir.2017.00086 PMC5671506 29163066

[brb32235-bib-0045] Zastrow, A., Kaiser, S., Stippich, C., Walther, S., Herzog, W., Tchanturia, K., Belger, A., Weisbrod, M., Treasure, J., & Friederich, H. C. (2009). Neural correlates of impaired cognitive‐behavioral flexibility in anorexia nervosa. American Journal of Psychiatry, 166(5), 608–616. 10.1176/appi.ajp.2008.08050775. 19223435

[brb32235-bib-0046] Zhang, S., Hu, S., Chao, H. H., Luo, X., Farr, O. M., & Li, C. S. (2012). Cerebral correlates of skin conductance responses in a cognitive task. Neuroimage, 62(3), 1489–1498. 10.1016/j.neuroimage.2012.05.036 22634217 PMC3408848

[brb32235-bib-0047] Zipfel, S., Giel, K. E., Bulik, C. M., Hay, P., & Schmidt, U. (2015). Anorexia nervosa: Aetiology, assessment, and treatment. Lancet Psychiatry, 2(12), 1099–1111. 10.1016/s2215-0366(15)00356-9. 26514083

