# Supplementary material for: Attenuated neuronal and autonomic responses during error processing in anorexia nervosa
Source: Brain Behav. 2021 Jul 28;11(8):e2235. doi: 10.1002/brb3.2235 (PMC8413769; doi:10.1002/brb3.2235)
Supplement: Supplementary file 1 — Suporting information [file BRB3-11-e2235-s001.docx]

**SUPPORTING INFORMATION**

**TABLE S1** Main effect of group during the Go/Nogo task

| Region of activation | Right/  Left | Brodmann Area | Cluster size | MNI coordinates | | | *F value* |
| --- | --- | --- | --- | --- | --- | --- | --- |
|  |  |  |  | x | y | z |  |
| Precuneus | R | 7 | 2686 | 6 | -55 | 48.5 | 27.79 |
| Precuneus | L | 7 |  | -1.5 | -61 | 51.5 | 24.04 |
| Anterior Insula | L | 13 | 2027 | -54 | -40 | 23 | 26.56 |
| Superior Temporal Gyrus | L | 22 |  | -61.5 | -40 | 11 | 22.41 |
| Superior Temporal Gyrus | L | 42 |  | -67.5 | -29.5 | 6.5 | 22.14 |
| Cerebellum | L |  | 31079 | -7.5 | -46 | -43 | 25.92 |
| Fusiform Gyrus | L | 37 |  | -39 | -58 | -14.5 | 25.86 |
| Caudate Nucleus | L |  |  | -7.5 | 8 | -2.5 | 24.49 |
| Inferior Occipital Gyrus | R | 19 | 992 | 37.5 | -80.5 | -1 | 19.43 |
| Inferior Occipital Gyrus | R | 18 |  | 37.5 | -88 | -8.5 | 16.71 |
| Fusiform Gyrus | R | 18 |  | 27 | -91 | -13 | 16.56 |
| Lentiform Nucleus | L |  | 1000 | -28.5 | -4 | -8.5 | 18.41 |
| Anterior Insula | L | 13 |  | -42 | -2.5 | 3.5 | 16.52 |
| Cerebellum | L |  |  | -24 | 21.5 | 5 | 15.53 |
| Supramarginal Gyrus | R | 40 | 1107 | 57 | -44.5 | 30.5 | 17.41 |
| Inferior Parietal Lobule | R | 40 |  | 43.5 | -37 | 50 | 13.66 |
| Superior Parietal Lobule | L | 7 | 1234 | -24 | -44.5 | 59 | 17.33 |
| Inferior Parietal Lobule | L | 40 |  | -37.5 | -44.5 | 44 | 17.02 |
| Anterior Cingulate Gyrus | M | 32 | 1279 | 0 | 35 | 15.5 | 17.17 |
| Anterior Cingulate Gyrus | R | 32 |  | 13.5 | 33.5 | 23 | 15.49 |
| Anterior Cingulate Gyrus | L | 32 |  | -10.5 | 35 | 6.5 | 12.78 |
| Middle Frontal Gyrus | L | 6 | 468 | -25.5 | -5.5 | 57.5 | 16.28 |
| Middle Temporal Gyrus | L | 21 | 446 | -61.5 | -13 | -7 | 13.92 |
| Inferior Temporal Gyrus | L | 20 |  | -49.5 | -26.5 | -13 | 11.44 |
| Middle Frontal Gyrus | L | 10 | 509 | -30 | 44 | 20 | 13.87 |
| Medial Frontal Gyrus | L | 9 |  | -22.5 | 36.5 | 23 | 11.19 |

*Note:* Maxima of regions showing significant BOLD activation when looking at the main effect of group at the whole‐brain level (voxel‐level p<0.005 uncorr., cluster‐level, p<0.05, FDR corr.); Abbreviations: L – left, R – right; M – middle.
